# A High-Salt Diet Exacerbates Liver Fibrosis through *Enterococcus*-Dependent Macrophage Activation

**DOI:** 10.1128/spectrum.03403-22

**Published:** 2023-02-14

**Authors:** Xujun Zhang, Yan Liang, Jingjing Jiang, Chong Lu, Fan Shi, Qingyi Cao, Yanhui Zhang, Hongyan Diao

**Affiliations:** a State Key Laboratory for Diagnosis and Treatment of Infectious Diseases, National Clinical Research Center for Infectious Diseases, National Medical Center for Infectious Diseases, Collaborative Innovation Center for Diagnosis and Treatment of Infectious Diseases, The First Affiliated Hospital, Zhejiang University School of Medicine, Zhejiang University, Hangzhou, China; b Jinan Microecological Biomedicine Shandong Laboratory, Jinan, China; c Key Laboratory of Aging and Cancer Biology of Zhejiang Province, School of Basic Medical Sciences, Hangzhou Normal University, Hangzhou, China; d Department of Nephrology, The First Affiliated Hospital of Wenzhou Medical University, Wenzhou, Zhejiang, China; e Department of Gastroenterology, Jinhua Hospital of Zhejiang University, Jinhua, Zhejiang, China; Jilin University

**Keywords:** *Enterococcus*, macrophage, high-salt diet, liver fibrosis

## Abstract

People consume more salt than the recommended levels due to poor dietary practices. The effects of long-term consumption of high-salt diets (HSD) on liver fibrosis are unclear. This study aimed to explore the impact of HSD on liver fibrosis. In this study, a carbon tetrachloride (CCL_4_)-induced liver fibrosis mouse model was used to evaluate fibrotic changes in the livers of mice fed a normal diet (ND) and an HSD. The HSD exacerbated liver injury and fibrosis. Moreover, the protein expression levels of transforming growth factor β (TGF-β), tumor necrosis factor alpha (TNF-α), and monocyte chemoattractant protein 1 (MCP-1) were significantly higher in the HSD group than in the normal group. The proportion of macrophages and activation significantly increased in the livers of HSD-fed mice. Meanwhile, the number of macrophages significantly increased in the small intestinal lamina propria of HSD-fed mice. The levels of profibrotic factors also increased in the small intestine of HSD-fed mice. Additionally, HSD increased the profibrotic chemokines and monocyte chemoattractant levels in the portal vein blood. Further characterization suggested that the HSD decreased the expression of tight junction proteins (ZO-1 and CLDN1), enhancing the translocation of bacteria. *Enterococcus* promoted liver injury and inflammation. *In vitro* experiments demonstrated that *Enterococcus* induced macrophage activation through the NF-κB pathway, thus promoting the expression of fibrosis-related genes, leading to liver fibrogenesis. Similarly, *Enterococcus* disrupted the gut microbiome *in vivo* and significantly increased the fibrotic markers, TGF-β, and alpha smooth muscle actin (α-SMA) expression in the liver.

**IMPORTANCE** This study further confirms that *Enterococcus* induce liver fibrosis in mice. These results indicate that an HSD can exacerbate liver fibrosis by altering the gut microbiota composition, thus impairing intestinal barrier function. Therefore, this may serve as a new target for liver fibrosis therapy and gut microbiota management.

## INTRODUCTION

A high-salt diet (HSD) increases the risks of various health complications, including chronic kidney disease, diabetes, cardiovascular diseases, and chronic inflammation (1, 2). According to several studies, an HSD is pro-inflammatory and can promote immunity. Many studies have also shown that excessive sodium intake can lead to autoimmune diseases (3). Animal studies have indicated that an HSD can adversely affect gut health by aggravating tissue inflammation and autoimmune diseases. An HSD boosts Th17 generation and exacerbates induced experimental autoimmune encephalomyelitis (4, 5). Recent research has also shown that an HSD can strengthen the intrarenal immune defense against pyelonephritis (6). Additionally, with an HSD, lymphoid tissues have a higher osmolality (7). However, the specific mechanisms of HSD interactions with the immune system are unclear.

Salt stress first affects the intestine. As a result, researchers have recently examined the effects of HSD on the intestinal microbiome. Earlier studies indicated that an HSD alters the composition of the intestinal flora in pigs (8). An HSD also alters the intestinal microbiome related to macrophages and Th17 cell responses (9). Moreover, Listeria monocytogenes causes systemic infections in HSD mice (6). Therefore, the intestinal flora is a potential target for treating many HSD-related diseases. However, only a few studies have assessed how HSD affects the intestinal flora.

The gut-liver axis connects the liver and intestine (10). Intestinal flora dysbiosis increases the influx of harmful substances into the liver through portal vein circulation (11). Liver fibrosis is a repair response to chronic liver injuries caused by various pathogens via complex cellular interactions (12). Hepatic stellate cells (HSC) are the primary target of fibrogenic stimuli in the diseased liver (13). Furthermore, distinct subsets of monocytes/macrophages and immune cells have fibrosis-promoting and inflammation-promoting properties in the liver (14, 15). Monocyte chemoattractant protein 1 (MCP-1) is a chemotactic cytokine regulating mononuclear inflammatory cell recruitment (16). Activated macrophages activate HSCs by synthesizing several cytokines, including transforming growth factor β (TGF-β), platelet-derived growth factor (PDGF), and tumor necrosis factor alpha (TNF-α) (17, 18). Clinical studies have shown that excess sodium chloride (NaCl) can increase the counts of monocytes in peripheral blood (19). However, the impact of an HSD on liver fibrosis is unclear.

In this study, a carbon tetrachloride (CCl_4_)-induced liver injury model was used to assess the effect of an HSD on liver fibrosis. The role of the intestinal microflora in liver damage was also assessed. Furthermore, the relationship between macrophages and fibrogenesis was assessed.

## RESULTS

### HSD exacerbates liver fibrosis.

**FIG 1:**
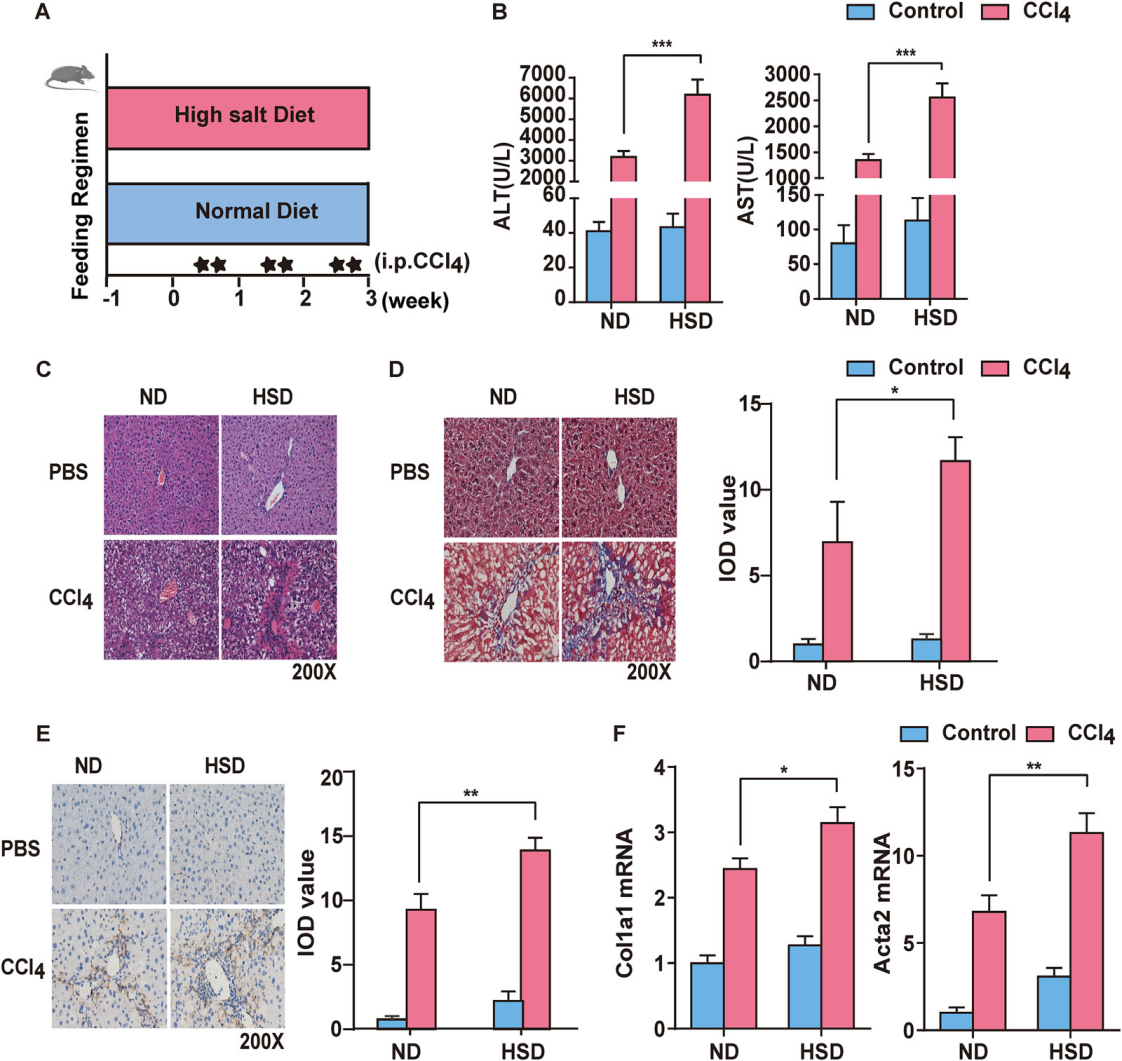
HSD exacerbates CCl_4_-induced liver fibrosis. (A) Schematic of feeding regimen in a murine model of liver fibrosis. (B) Detection of serum biochemical AST and ALT levels. (C) H&E staining of liver tissue slices (200×) (D) Liver fibrosis detected using Masson staining (left). Integrated optical density (IOD) analyzed using Image-Pro Plus 6 (right). (E) Immunohistochemistry (IHC) of α-SMA (left). IOD analyzed using Image-Pro Plus 6 (right). (F) Relative expression of collagen I (Col1a1) and α-SMA (ACTA2) mRNA in liver. Results are expressed as mean ± SEM. i.p., intraperitoneal. *, *P* < 0.05; **, *P* < 0.01; ***, *P* < 0.001; ****, *P* < 0.0001, compared to the ND group.

A murine model of CCl_4_-induced liver fibrosis was established to explore the impact of a normal diet (ND) and a high-salt diet (HSD) on hepatic fibrosis. Food intake was monitored for 4 weeks (Fig. 1A). Aminotransferase (ALT), aspartate aminotransferase (AST), and hyaluronic acid (HA) indices were used to evaluate liver injury. The HSD significantly increased the activities of ALT, AST, and HA (Fig. 1B; see Fig. S1 in the supplemental material). Hematoxylin and eosin (H&E) staining showed that the HSD resulted in massive steatosis and inflammatory infiltration in the livers of mice compared with the ND group (Fig. 1C). Masson staining showed that the HSD significantly increased liver fibrosis (Fig. 1D). Furthermore, the HSD increased the levels of inflammatory cytokines and indicators of liver fibrosis (alpha smooth muscle actin [α-SMA], collagen I, TGF-β, tissue inhibitor matrix metalloproteinase 1 [TIMP-1], and PDGF) (Fig. 1E and F; Fig. S2). Taken together, these results show that an HSD aggravates inflammation and fibrosis in liver injury.

### HSD regulates liver macrophage activation during liver fibrosis.

**FIG 2:**
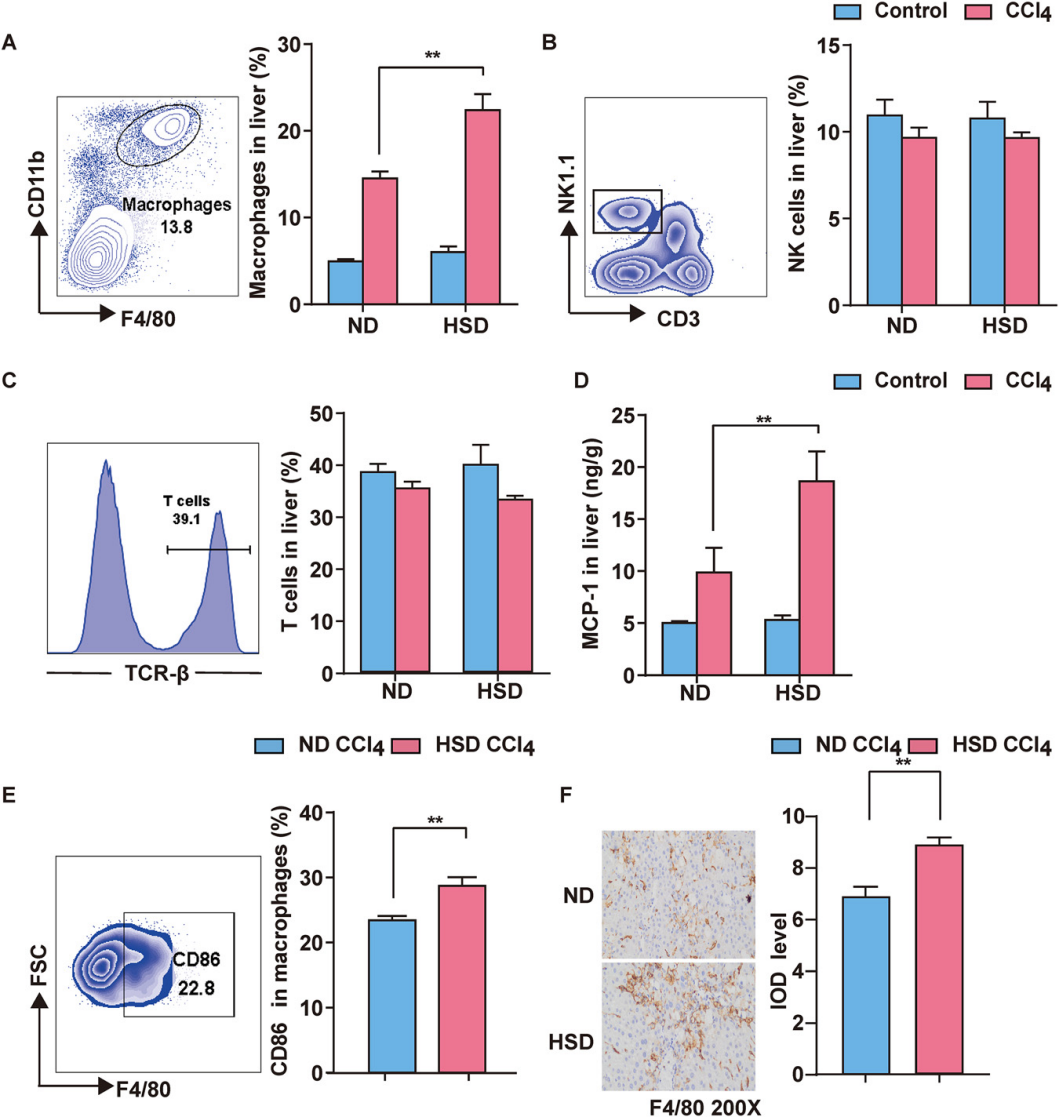
HSD governs liver macrophage activation during liver fibrosis. (A) Representative flow cytometry images of liver-infiltrating monocytes (left). Bar plots showing the percentage of macrophages in the liver (right). (B) Representative flow cytometry images of liver NK cells (left). Bar plots showing the percentage of NK cells in the liver (right). (C) Representative flow cytometry images of T cells (left). Bar plots showing the percentage of T cells in the liver (right). (D) MCP-1 content in the liver, determined using ELISA. (E) Representative flow cytometry images of CD86 expression in macrophages (left). Bar plots showing the percentage of CD86 expression in macrophages (right). (F) IHC of macrophages; F4/80 staining. Results are expressed as mean ± SEM. *, *P* < 0.05; **, *P* < 0.01; ***, *P* < 0.001; ****, *P* < 0.0001, compared to the ND group.

Multiple studies have shown that immune cells play a crucial role in liver inflammation and fibrosis development. Moreover, the aggravation of hepatic fibrosis and immune balance are closely related. Herein, the effect of an HSD was further analyzed to identify the different immune subsets. The HSD increased macrophage infiltration into the liver (Fig. 2A). However, the HSD did not significantly affect NK and T cells (Fig. 2B and C). Monocyte chemotactic protein 1 (MCP-1) is a chemotactic agent for macrophages. Herein, the HSD released more MCP-1 in the liver than in the ND group (Fig. 2D). However, further studies should evaluate macrophage phenotypes and function. The surface expression of the costimulatory molecules CD80 and CD86 was determined after gating F4/80^+^ CD11b^+^ macrophage. These molecules significantly exacerbated inflammation (Fig. 2E; see Fig. S3 in the supplemental material). Macrophage-specific F4/80 immunohistochemistry analysis (Fig. 2F) showed an increased F4/80 immunostaining around the liver of the HSD group. These results show that an HSD causes an immune imbalance in the liver due to the increased macrophage activation, as indicated by immune cell expression in whole liver tissue.

### HSD modulates intestinal barrier function and inflammation.

**FIG 3:**
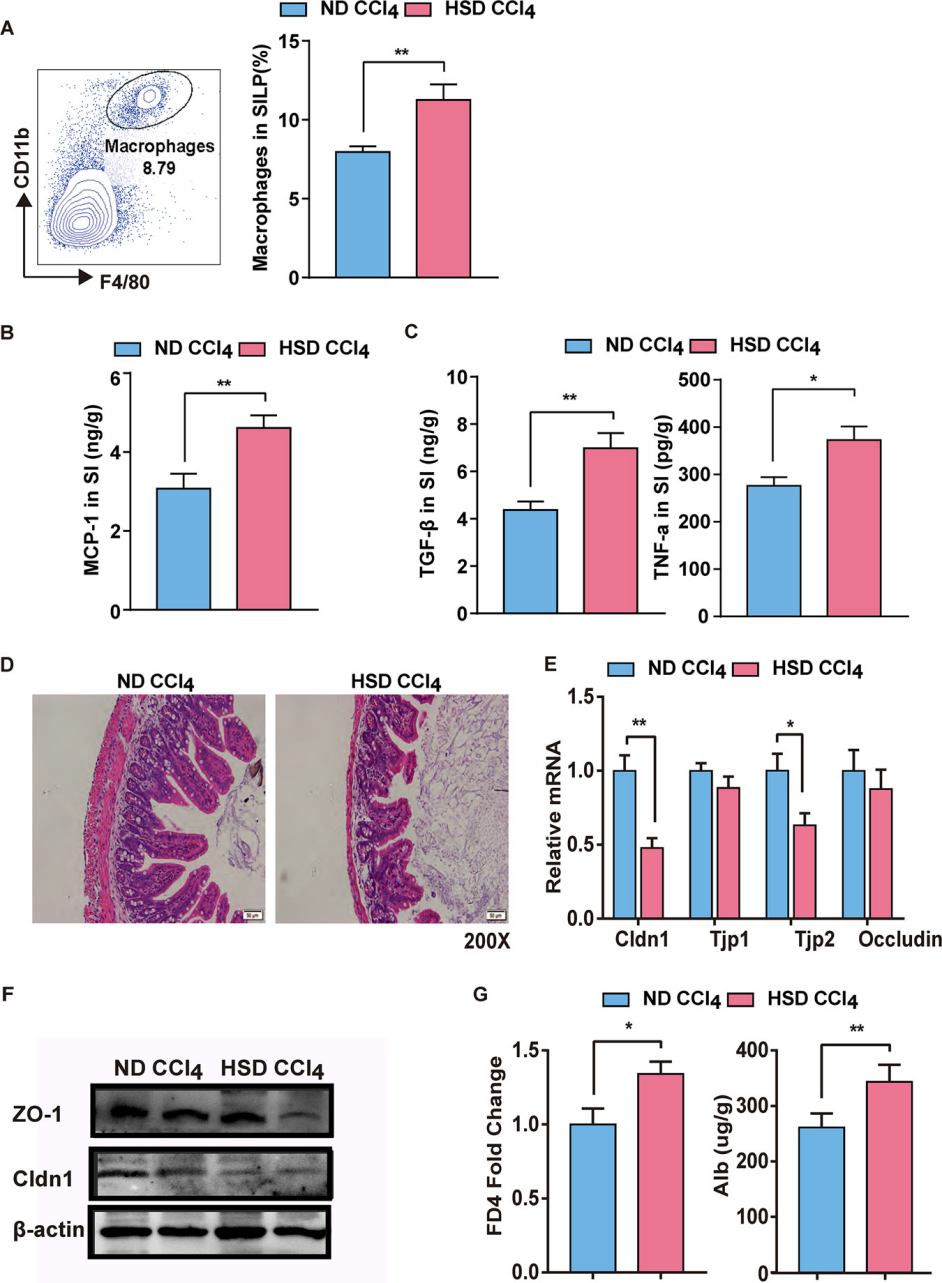
HSD-modulated disruption of intestinal barrier function and inflammation. (A) Representative flow cytometry images of macrophages in small intestine lamina propria (SILP) (left). Bar plots showing the percentage of macrophages in small intestine lamina propria (right). (B) MCP-1 content in small intestine lamina propria, determined using ELISA. (C) TGF-β and TNF-α contents in small intestine lamina propria, determined using ELISA. (D) Representative image of an H&E-stained section of intestine. (E) mRNA expression of tight junction proteins in the intestine. (F) Protein expression of tight junction proteins in the intestine. (G) Serum FD4 levels (left). Alb concentration in feces (right). Results are expressed as mean ± SEM. *, *P* < 0.05; **, *P* < 0.01; ***, *P* < 0.001; ****, *P* < 0.0001, compared to the ND group.

Diet regulates the intestinal immune response. Herein, HSD significantly modulated the host intestinal immune system of the mice. Quantification of flow cytometry analysis of isolated lamina propria lymphocytes indicated macrophage infiltration and no significant changes in the T-cell subset (Fig. 3A and Fig. S4). However, such effects were not observed in the colonic lamina propria (Fig. S5). The HSD also released more MCP-1, TGF-β, and TNF-α in the lamina propria lymphocytes than in the ND group (Fig. 3B, C). H&E staining demonstrated that the HSD caused a significant intestinal injury (Fig. 3D). Gene and protein expressions of intestinal tight junction proteins as a marker of barrier integrity were also measured. Low expression decreased intestinal barrier integrity in the HSD group (Fig. 3E and F). The HSD group had increased serum levels of FD4 and fecal albumin compared with the ND group, indicating that the HSD increased intestinal permeability (Fig. 3G). These results indicate that an HSD can destroy the balance of immune responses and disrupt intestinal barrier function.

### HSD significantly increases *Enterococcus* abundance in the intestine.

**FIG 4:**
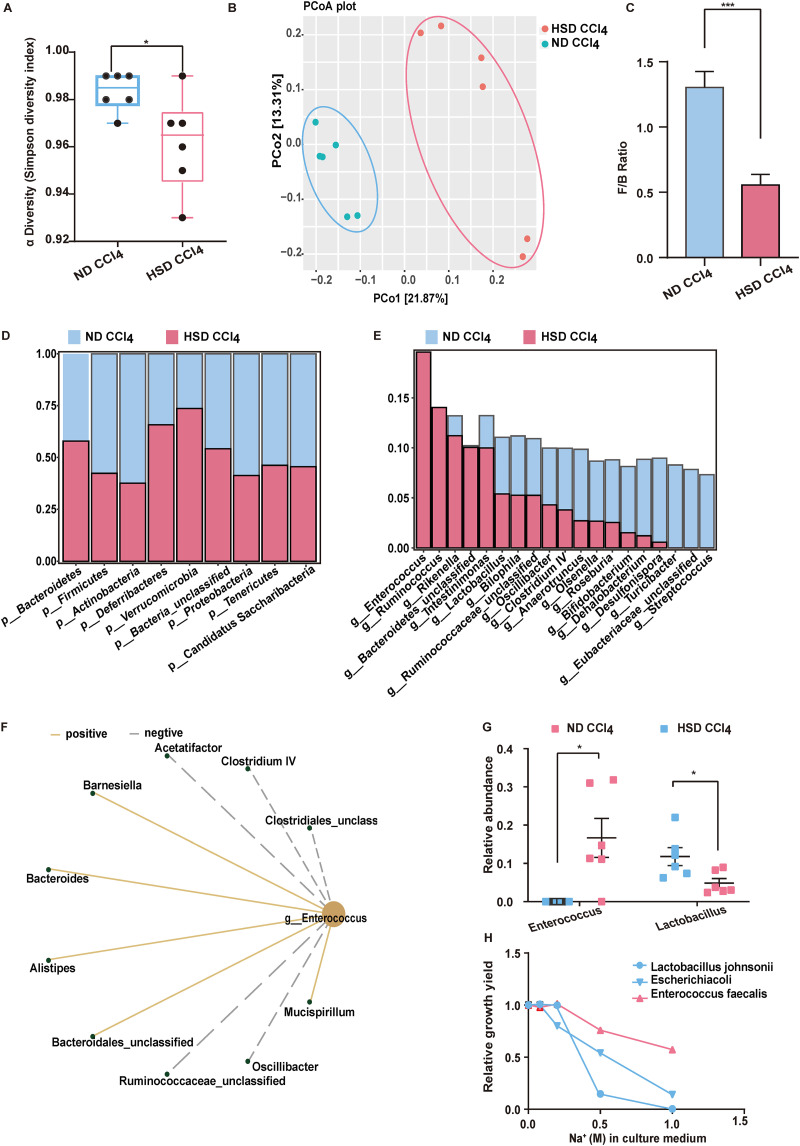
HSD mediates regulation of intestinal flora. (A) α-diversity analyses of intestinal flora. (B) Principal coordinates analysis (PCoA) of the samples. (C) *Firmicutes*-to-*Bacteroidetes* ratio. (D) The relative abundance of intestinal microbiota at the phylum level (nine most relatively abundant). (E) Differential bacteria at the genus level. (F) Relationship between *Enterococcus* and other differential bacteria at the genus level. (G) Relative abundance of *Enterococcus* and *Lactobacillus*, determined using qPCR. (H) Tolerance of different bacteria to salt concentrations. Results are expressed as mean ± SEM. *, *P* < 0.05; **, *P* < 0.01; ***, *P* < 0.001; ****, *P* < 0.0001, compared to the ND group.

Several studies have shown that diet can alter the composition of intestinal flora. Herein, the fecal microbiota was analyzed using the 16S rDNA sequence. The Simpson index was used to cluster the fecal microbiome into ND CCL_4_ and HSD CCL_4_. The HSD significantly affected the mucosal microbiota composition in the HSD group compared to the ND group (Fig. 4A). The HSD significantly changed the bacterial flora, mainly indicating that the principal-coordinate analysis (PCoA) separated the samples based on the presence or absence of high salt in the diet (Fig. 4B). Moreover, the HSD modulated the intestinal microbiota in the feces at the phylum level (Fig. 4D). The proportion of *Firmicutes* increased in the HSD CCL_4_ samples compared to the ND CCL_4_ samples. In contrast, the proportion of *Bacteroides* decreased in the HSD CCL_4_ samples compared to the ND CCL_4_ (Fig. 4C). The top 20 core communities at the genus level also had some differences. A Kruskal-Wallis test showed that the HSD was correlated with the relative abundance of 19 genera, positively correlated with the relative abundance of *Enterococcus*, *Bacteroidetes*, and *Ruminococcus*, and negatively correlated with the relative abundance of 14 genera, including *Lactobacillus*, *Oscillibacter*, and *Turicibacter* (Fig. 4E and F). Quantitative PCR (qPCR) showed that the HSD increased *Enterococcus* abundance and significantly decreased the *Lactobacillus* levels (Fig. 4G). The linear discriminant analysis (LDA) effect size (LEfSe) was determined at multiple phylogenetic levels to identify differentially expressed microbial biomarkers. The characteristic biomarkers that differed between the ND CCL_4_ and HSD CCL_4_ groups are shown in Fig. S6. Furthermore, *Enterococcus* spp. had better tolerance to NaCl *in vitro* than the common bacteria Escherichia coli and Lactobacillus johnsonii (Fig. 4H). These results suggest that an HSD alters the intestinal flora composition, especially *Enterococcus* abundance.

### *Enterococcus* induces the activation of macrophages and enhances intestinal permeability.

**FIG 5:**
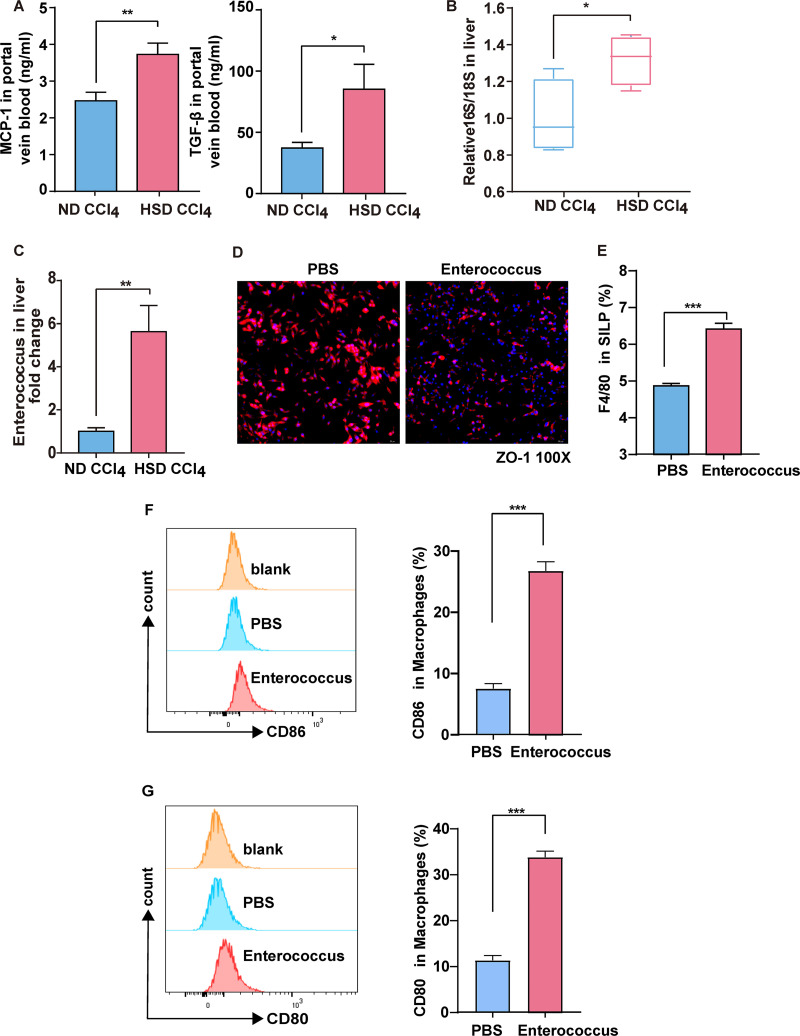
*Enterococcus* primes the activation state of macrophages and enhanced intestinal permeability. (A) Plasma MCP-1 and TGF-β concentrations in the portal veins. (B) qPCR data for the 16S-to-18S ratio in liver. (C) qPCR data for the relative abundance of *Enterococcus* in liver. (D) Immunofluorescence staining for zonula occludens 1 (ZO-1). (E) Change in the F4/80 ratio of the SILP after *Enterococcus* stimulation. (F) Change in the CD86 ratio of the macrophages after *Enterococcus* stimulation. (G) Change in the CD80 ratio of macrophages after *Enterococcus* stimulation. Results are expressed as mean ± SEM. *, *P* < 0.05; **, *P* < 0.01; ***, *P* < 0.001; ****, *P* < 0.0001, compared to the ND group.

The liver-gut axis is associated with intestinal microbial dysbiosis. Herein, the HSD increased MCP-1 and TGF-β levels in the portal vein (Fig. 5A). Increased intestinal permeability may induce bacterial translocation, leading to bacterial migration from the gut to the liver. Bacterial translocation into the mesenteric lymph nodes (MLN) and liver, assessed using the tissue culture method, is shown in Table 1. The HSD CCl_4_ group had more colonies than the ND CCl_4_ group. The HSD significantly increased the content of total bacteria in the liver compared with that in the ND group (Fig. 5B). Additionally, *Enterococcus* abundance was higher in the HSD CCl_4_ group than in the other samples (Fig. 5C). *In vitro* experiments were performed to determine the effect of *Enterococcus* on the intestinal barrier. *Enterococcus* significantly decreased the ZO-1 and CLDN1 levels, indicating an intestinal barrier disruption *in vitro* (Fig. 5D; Fig. S7). Flow cytometry was used to analyze the effect of *Enterococcus* coculture on macrophages. *Enterococcus* promoted the activation of macrophages in intestinal lamina propria lymphocytes (Fig. 5E) and the expression of the surface activation markers CD80 and CD86 in macrophages (Fig. 5F and G). Furthermore, MCP-1 levels were increased in the culture supernatant compared with the macrophage treated with phosphate-buffered saline (PBS) (Fig. S8). These results indicate that *Enterococcus* can disrupt the intestinal barrier and activate macrophages.

**TABLE 1:** Bacterial translocation of the mesenteric lymph nodes and liver

Group	Bacterial translocation (mean ± SEM [%])	
	Liver	MLN^*a*^
ND CCl_4_	23.33 ± 3.33	43.75 ± 7.46
HSD CCl_4_	50 ± 5.77	83.33 ± 16.67

a MLN, mesenteric lymph nodes.

### *Enterococcus*-infected macrophages activate hepatic stellate cells through the NF-κB signaling pathway.

**FIG 6:**
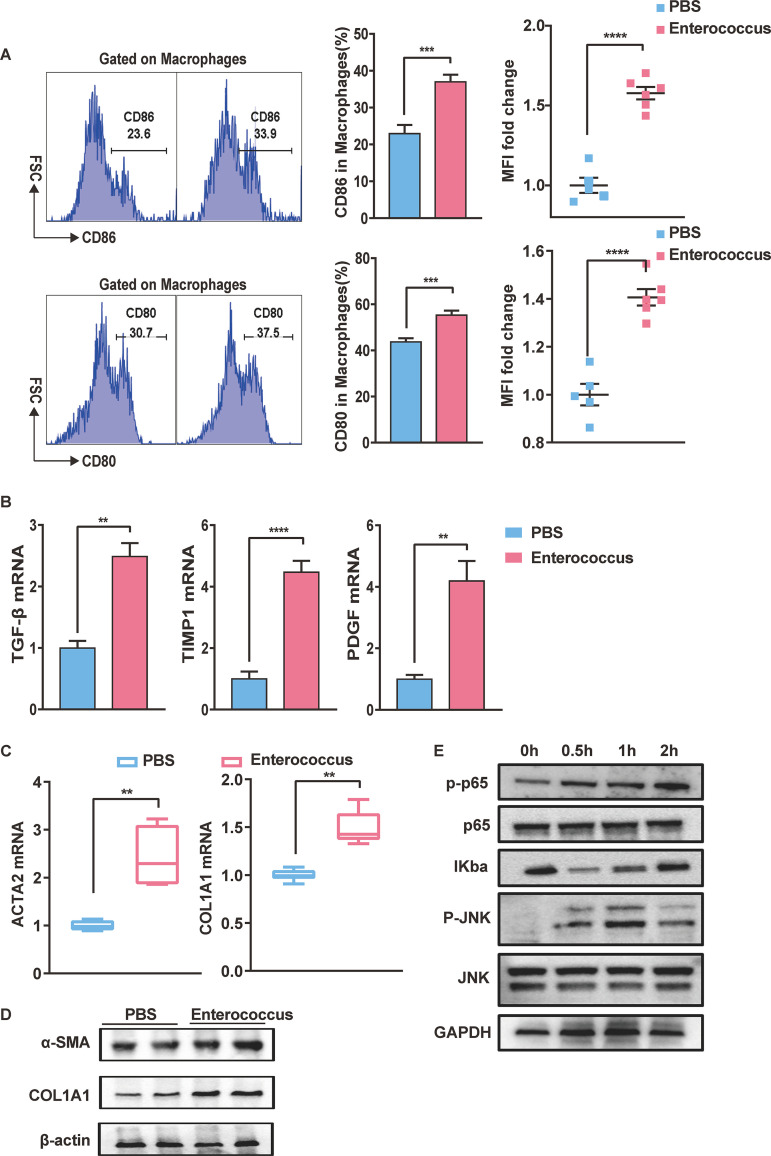
*Enterococcus*-induced activation of macrophages and hepatic stellate cells through the NF-κB signaling pathway. (A) Changes in the CD86 (top) and CD80 (bottom) ratios in the primary hepatocytes and lymphocytes stimulated by *Enterococcus*. (B) TGF-β, TIMP-1, and PDGF production upon uptake into macrophages after *Enterococcus* stimulation. (C and D) mRNA and protein expression of collagen I (COL1A1) and α-SMA (ACTA2) levels in an *in vitro* coculture system. (E) Activated canonical nuclear factor (NF)-kappa B pathway at different time points after *Enterococcus* treatment. Results are expressed as mean ± SEM. *, *P* < 0.05; **, *P* < 0.01; ***, *P* < 0.001; ****, *P* < 0.0001, compared to the PBS group. MFI, mean fluorescence intensity.

The role of *Enterococcus* in the development of liver fibrosis was also investigated. Previous studies have shown that bacteria or bacterial products can enhance the activation of immune cells. Herein, *Enterococcus*-infected primary macrophages expressed more CD80 and CD86 costimulatory molecules in the liver than macrophages from the PBS group (Fig. 6A). Moreover, TGF-β, TIMP-1, and PDGF secretion increased after *Enterococcus* stimulation (Fig. 6B). Macrophage activation plays a key role in liver fibrosis. The function of activated macrophages in regulating HSC was assessed using an *in vitro* coculture system. Collagen I and α-SMA were upregulated in the coculture system at the mRNA and protein levels (Fig. 6C). The Toll-like receptor (TLR)-mediated signaling pathway is critical for host defense against invading pathogens (20). The expression levels of TLRs in the liver were analyzed to determine which TLR changed most obviously. Immunoblotting was performed to assess protein levels. These results revealed that there was an increase in TLR2 protein, and TLR5 levels were not elevated (Fig. S9). Gram-positive *Enterococcus* promoted inflammation by acting on TLR2, consistent with existing studies (21). *Enterococcus* stimulates macrophage activation by promoting the activation of the NF-κB pathway (Fig. 6D). Furthermore, the levels of the inflammatory factors TNF-α and interleukin 1B (IL-1B) and the phospho p65 (P-p65) were inhibited after macrophages were pretreated with the TLR2 inhibitor C29 (Fig. S10). Thus, *Enterococcus* plays a major role in promoting inflammatory processes, which are a major cause of liver fibrosis.

### *Enterococcus* promotes liver fibrosis.

**FIG 7:**
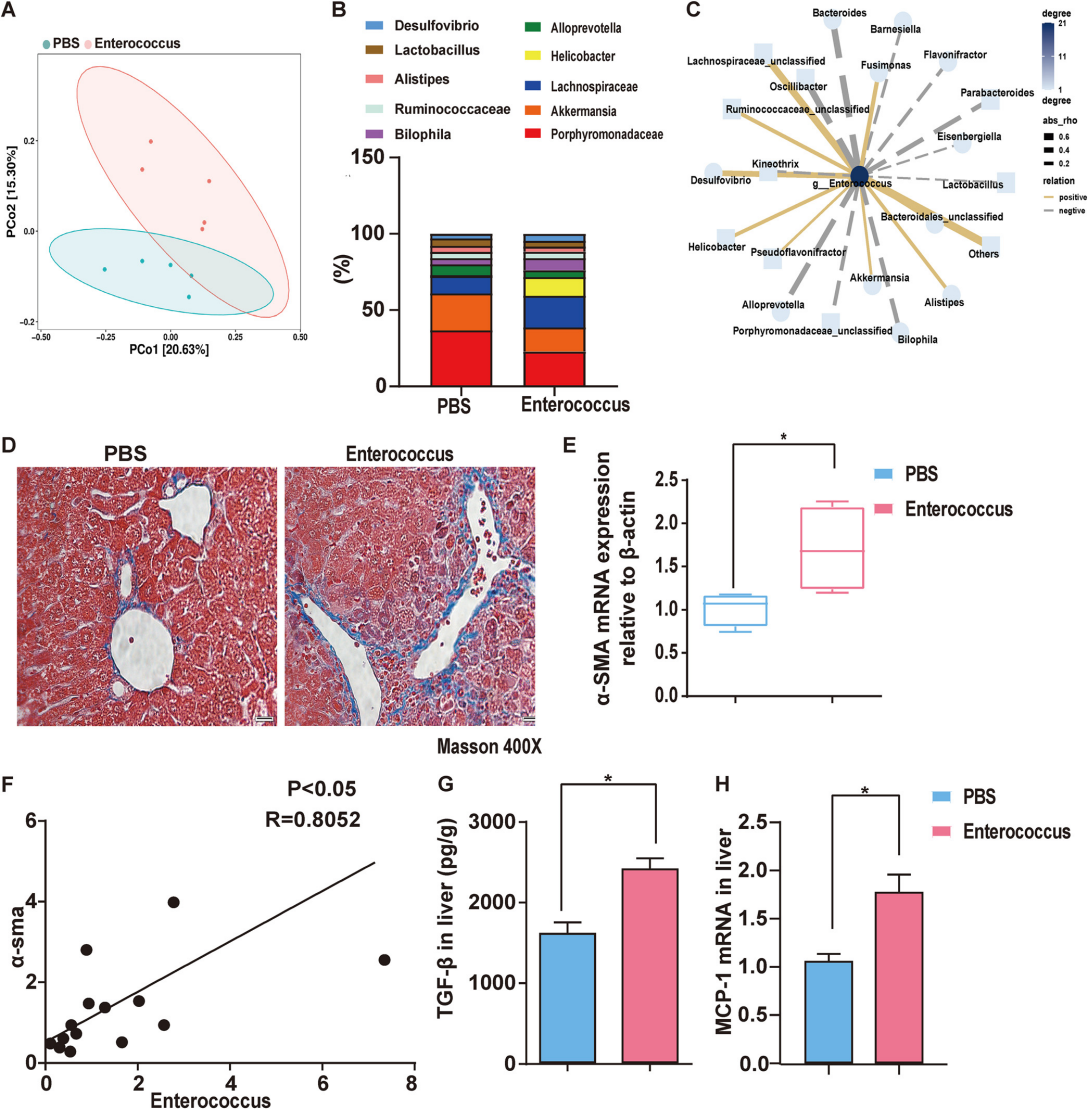
*Enterococcus* promotes liver fibrosis in mice. (A) Principal coordinates analysis (PCoA) of the samples. (B) Relative abundance of intestinal microbiota at the phylum level. (C) Relationship between *Enterococcus* and other differential bacteria at the genus level. (D) Liver fibrosis in mice was detected using Masson staining. (E) qPCR data for the relative abundance of α-SMA in liver. (F) Correlation between α-SMA and *Enterococcus* expression. (G) ELISA data for the relative abundance of TGF-β in liver. (H) qPCR data for the relative abundance of MCP-1 in liver. Results are expressed as mean ± SEM. *, *P* < 0.05, **, *P* < 0.01, ***, *P* < 0.001, ****, *P* < 0.0001, compared to the PBS group.

Herein, the HSD regulated the gut microbiome in CCl_4_-induced liver fibrosis. The mice were inoculated with an equal amount of PBS and *Enterococcus* cells to further evaluate the role of an *Enterococcus*-regulated microbiome in CCl_4_-induced liver fibrosis, with continuous infusion of the bacterium after 1 week of bacterium injections. The structure of the intestinal flora of the *Enterococcus* recipients was different from that of the control (Fig. 7A). The top 10 flora at the genus level are shown in Fig. 7B. *Porphyromonadaceae*, *Akkermansia*, *Alloprevotella*, and *Lactobacillus* were significantly downregulated, while *Desulfovibrio*, *Bilophila*, *Helicobacter*, and *Lachnospiraceae* were upregulated. The *Enterococcus* content in feces was significantly higher in the modeling group than in the control group (Fig. S11), leading to flora changes (Fig. 7C). To further investigate changes in the liver, *Enterococcus* was significantly increased (Fig. S12). Masson staining showed that the liver fibrosis grew more severe in the *Enterococcus* group (Fig. 7D). Moreover, the liver injury increased the release of ALT and AST (Fig. S13A). The relation of α-SMA levels to the expression of Enterococcus faecalis was further explored to prove that *Enterococcus* aggravated liver fibrosis (Fig. 7E and F). The liver expressions of pro-inflammatory (MCP-1) and profibrosis (TGF-β) factors were evaluated to further explore the mechanisms of *Enterococcus*-induced liver fibrosis (Fig. 7G and H; Fig. S13B). These results indicate that *Enterococcus* promotes liver fibrosis in mice.

## DISCUSSION

Previous studies have shown that inflammation promotes CCl_4_-induced liver fibrosis development and regression (22). In addition to host genetic makeup, other factors, such as diet and microbiota composition, affect the development of inflammatory diseases (23, 24). In this study, HSD aggravated liver fibrosis in mice by dysregulating the balance of the intestinal flora, increasing the harmful bacteria, and promoting macrophage infiltration. This experiment may provide a basis for liver fibrosis therapy.

However, there is growing concern over the harm of HSD. Previous studies only focused on heart disease, stroke, and hypertension (25). Mounting evidence has shown that excessive dietary salt intake induces an inflammatory response. Furthermore, the severity of liver fibrosis is associated with macrophage infiltration in mice fed an HSD, consistent with previous literature. Some studies have shown that macrophages promote hepatic fibrosis by activating NF-κB in hepatic stellate cells (26). Herein, the HSD increased the activity and production of TGF-β, MCP-1, and TNF-α in lamina propria macrophages. Moreover, diet changes altered the intestinal tract microflora. HSD-induced gut dysbiosis increased the abundance of the phylum *Bacteroides* and reduced the abundance of the phylum *Firmicutes*. Previous work on alcoholic liver disease and early kidney injury caused by HSD has shown similar results (27–29). Moreover, HSD increased the relative abundance of *Enterococcus*, *Bacteroidetes*, and *Ruminococcus* at the genus level, while reducing *Lactobacillus* abundance, consistent with earlier studies. Notably, *Enterococcus* was positively correlated with liver severity, indicating that *Enterococcus* abundance may be associated with increased severity of liver fibrosis. Interestingly, the results show that *Enterococcus* could affect the composition and abundance of gut flora, thus promoting liver injury in mice.

Our understanding of the gut-liver axis has improved in the last decade (30). Microbial dysbiosis causes mucosal inflammation, impaired barrier function, and gut permeability (31–33). Increased intestinal permeability enhances the translocation of microorganisms, inflammatory factors, and metabolites into the systemic circulation via the portal vein (34). An alcoholic fatty liver study showed that *Enterococcus* translocation from the gut into the liver can aggravate liver injury (35). Another study reported that an HSD could cause kidney damage due to intestinal barrier destruction and intestinal bacterial translocation (29). In this study, the HSD increased gut permeability and altered the gut barrier, characterized by disruption of the tight junction proteins. Therefore, further research should assess whether *Enterococcus* translocation can lead to liver injury in mice fed an HSD. Herein, *Enterococcus* promoted macrophage activation and induced the activation and proliferation of HSCs, which are key in the development of liver fibrosis. *In vitro* results showed that *Enterococcus* induced macrophage activation by activating the TLR2-NF-κB signaling pathway and secreting inflammatory cytokines. *Enterococcus* cells were also detected in the mesenteric lymph node and liver. An *in vivo* experiment confirmed these results. The HSD changed the gut flora, enhancing macrophage activation via *Enterococcus*, thus exacerbating inflammation and fibrosis in mice.

**FIG 8:**
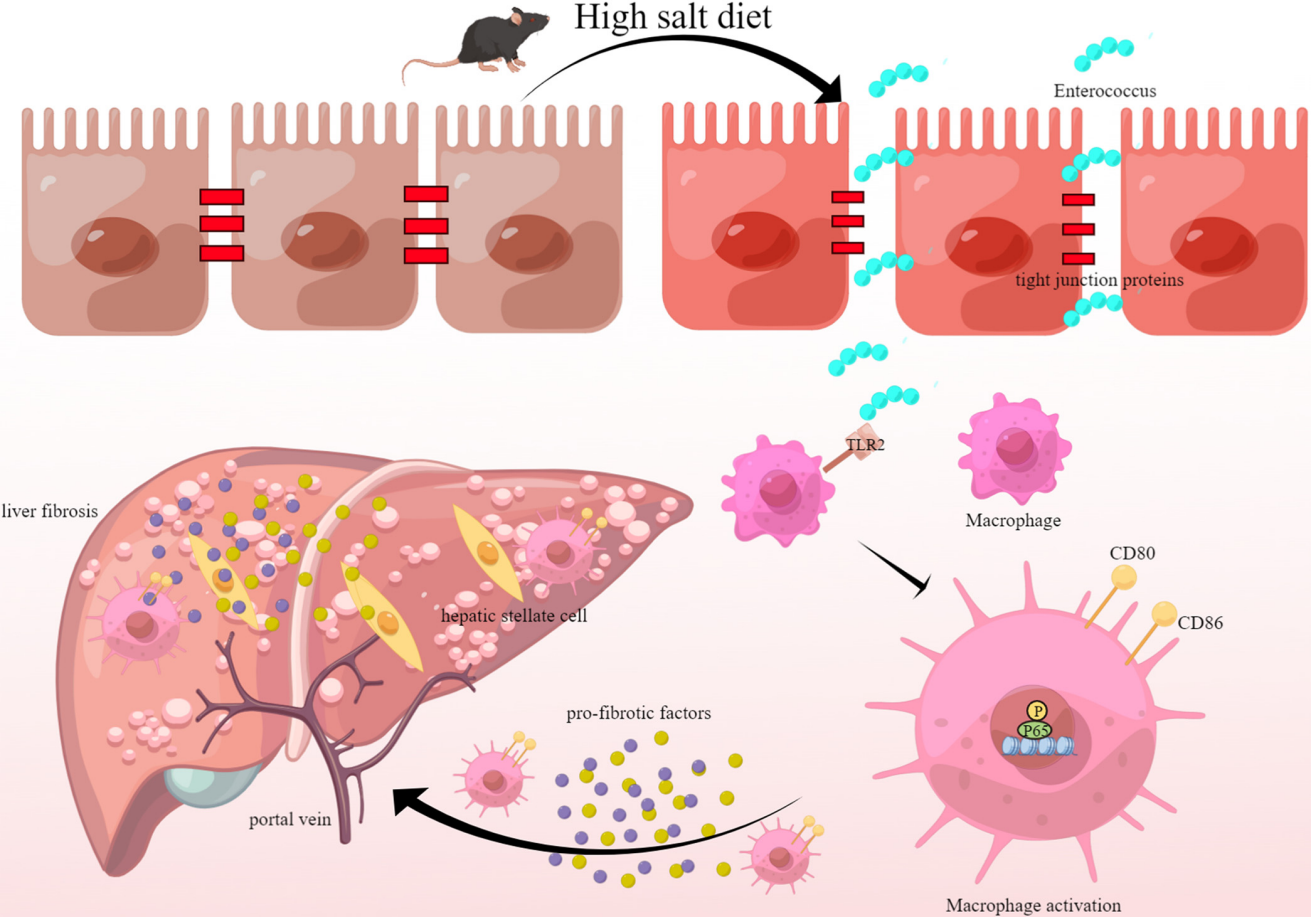
Molecular model depicting the mechanism of high-salt diet exacerbation of liver fibrosis through *Enterococcus*-dependent macrophage activation. An HSD is significantly positively correlated with the proportion of macrophages in the liver and the disruption of intestinal barrier function. *Enterococcus* induced macrophage activation through the NF-κB pathway, thus promoting the expression of fibrosis-related genes and leading to liver fibrogenesis.

In conclusion, the HSD aggravated liver fibrosis in mice. HSD is significantly positively correlated with the proportion of macrophages in the liver. Changes in the abundance of *Enterococcus* are closely related to the number of macrophages (Fig. 8). Healthy diets have attracted more attention in recent years. Therefore, these findings may guide healthy dietary habits and the treatment of liver fibrosis. This study provides a new perspective on the role of *Enterococcus* in liver and liver disease prevention. This study also provides insights into fecal transplantation and the use of probiotics. Furthermore, this study provides an experimental basis for further analysis of the altered gut flora that can activate the immune system. The bacterial infection is probably the beginning of a promiscuous response that leads to immunity, which has also been investigated in previous studies (36). Moreover, cytokines play a prominent role in multiple inflammatory diseases that cannot be ignored (37, 38). Various factors are involved in the pathogenesis of liver fibrosis. However, further research should investigate the relationship between HSD and the immune system. Furthermore, it is unknown whether other immune-related cells or proteins may play a similar role in hepatic damage.

## MATERIALS AND METHODS

### Mice.

All experimental procedures were approved by the Animal Care Ethics Committee of the First Affiliated Hospital, Zhejiang University (permit number 202107). Specific pathogen-free (SPF) female wild-type (WT) C57BL/6 (4-week-old) mice were purchased from the Laboratory Animal Center of Shanghai SLRC Experimental Animal Company Ltd. (Shanghai, China). The mice received normal chow and water *ad libitum* (ND) or a sodium-rich chow containing 4% NaCl and water containing 1% NaCl *ad libitum* (HSD) (39). For the induction of carbon tetrachloride (CCl_4_)-mediated progressive liver fibrosis, 6-week-old male C57BL/6J mice received intraperitoneal injections of CCl_4_ (two injections per week of 25% CCl_4_ solution in olive oil, 2 mL/kg body weight). For the induction of liver inflammation, CCl_4_ was administered (once), and the mice were killed 24 h later (“CCl_4_ once group”). Serum and liver tissue were stored at −80°C for further use. A piece of each liver was fixed with formaldehyde for histology.

### Bacterial colonization.

Five-week-old WT C57BL/6 mice female were acclimatized for 1 week and randomly divided into 2 groups: (i) the ND-CCl_4_ group, fed with a standard chow diet and PBS (0.2 mL/day) for a week before induction of CCl_4_-mediated progressive liver fibrosis as above, and (ii) the *Enterococcus*-CCl_4_ group, fed with a standard chow diet and *Enterococcus* (0.2 mL/day) for a week before induction of CCl_4_-mediated progressive liver fibrosis as above.

### Histological analyses.

To analyze morphological changes, liver and intestine samples were paraffin embedded, sectioned, and stained with hematoxylin and eosin (H&E) or Masson trichrome (MT). Immunohistochemical detection of proteins was performed.

### Liver function tests.

Blood samples from the inferior vena cava were centrifuged (3,000 × g for 10 min at 4°C) to segregate the serum or plasma. The serum or plasma was stored at –40°C for further analysis. The concentrations of ALT, AST, and H&E in the serum were determined using a 7600 analyzer (Hitachi High-Technologies Corporation, Tokyo, Japan).

### Cell isolation and flow cytometry.

Lamina propria lymphocytes from the small intestine were isolated as described. Liver-infiltrating leukocytes were isolated as previously described. The antibodies used in this study, including data, were determined on a FACSCanto II flow cytometer (BD Biosciences) and analyzed using FlowJo (Tree Star) or BD FACS Diva (BD Biosciences) software.

### Cell culture and bacterial culture.

LX-2, HT-29, and THP-1 cells were obtained from the American Type Culture Collection (ATCC; Manassas, VA). Cells were cultured in RPMI 1640 medium (Gibco) supplemented with 10% fetal bovine serum (FBS) (Gibco), 100 U/mL penicillin, and 100 mg/mL streptomycin. C29 was purchased from Med Chem Express (NJ, USA). In this study, the most common clinical strain of *Enterococcus* was used (40, 41). Enterococcus faecalis was grown in LB agar powder (Sango Biotech) in a shaker at 37°C for 8 h.

### Western blot analysis.

Western blot analysis was employed to assess protein expression. For Western blotting, antibodies to the following proteins were used: α-SMA (9245; Cell Signaling Technology [CST]); COL1A1 (72026; CST); P-p65 (3033; CST); p65 (8242; CST); IκBα (4814; CST); p-JNK (9255S; CST); JNK (9252; CST); TLR2 (Proteintech); TLR5 (Proteintech).

### Cytokine analysis.

TNF-α, MCP-1, and transforming growth factor β (TGF-β) levels were measured using human enzyme-linked immunosorbent assay (ELISA) Ready-Set-Go kits (eBioscience, San Diego, CA) according to the manufacturer’s instructions. The cytokine content was expressed as the amount per milliliter of plasma or supernatant.

Total RNA was isolated using TRIzol (TaKaRa), and cDNA was synthesized using a PrimeScript RT master mix (TaKaRa) according to the manufacturer’s instructions. Gene expression was quantified using the comparative threshold cycle (*C_T_*) method, with *C_T_* values normalized to β-actin. PCR was performed using SYBR premix Ex Taq II (TaKaRa) with specific primers.

### 16S sequencing analysis.

The total DNA was eluted in 50 μL of elution buffer and stored at –80°C until measurement in the PCR by LC-Bio Technology Co., Ltd. (Hangzhou, Zhejiang Province). The variable region of 16S rDNA (V3 plus V4) was amplified using the primers 341F (5′-CCTACGGGNGGCWGCAG-3′) and 805R (5′-GACTACHVGGGTATTCTAATCC-3′), which were tagged with a specific barcode per sample. The PCR products were purified using AMPure XT beads (Beckman Coulter Genomics, Danvers, MA, USA) and quantified using Qubit (Invitrogen, USA). The amplicon pools were prepared for sequencing, and the size and quantity of the amplicon library were assessed using a 2100 Bioanalyzer (Agilent, USA) and the library quantification kit for Illumina (Kapa Biosciences, Woburn, MA, USA), respectively. The libraries were sequenced on the HiSeq platform in paired-end 2 × 150-bp (PE150) format. The Chao1, Shannon, and Simpson indices were calculated using QIIME2.

### Statistical analysis.

GraphPad Prism 7 was used for statistical analyses. All data are expressed as mean ± standard error of the mean (SEM). A two-way analysis of variance (ANOVA), *t* test (and nonparametric tests), or Mann-Whitney test was performed to determine significance. For correlation analyses, Spearman’s rank correlation test was used. Differences were considered significant at *P* < 0.05. Fig. 8 (ID, IPSYAc8c5f) was drawn using the online plotting tool Figdraw.

### Ethics statement.

All experimental procedures were approved by the Animal Care Ethics Committee of the First Affiliated Hospital, Zhejiang University (permit number 202107), and all procedures were performed according to the *Guide for the Care and Use of Laboratory Animals* (42).

### Data availability.

The 16S rRNA gene sequencing data have been uploaded to the Sequence Read Archive (SRA) (accession no. PRJNA924053).
