# Supplementary material for: A High-Salt Diet Exacerbates Liver Fibrosis through Enterococcus-Dependent Macrophage Activation
Source: Microbiol Spectr. 2023 Feb 14;11(2):e03403-22. doi: 10.1128/spectrum.03403-22 (PMC10100947; doi:10.1128/spectrum.03403-22)
Supplement: supplemental file 1 — Fig. S1 to S13. Download spectrum.03403-22-s0001.pdf, PDF file, 4.0 MB [file spectrum.03403-22-s0001.pdf]

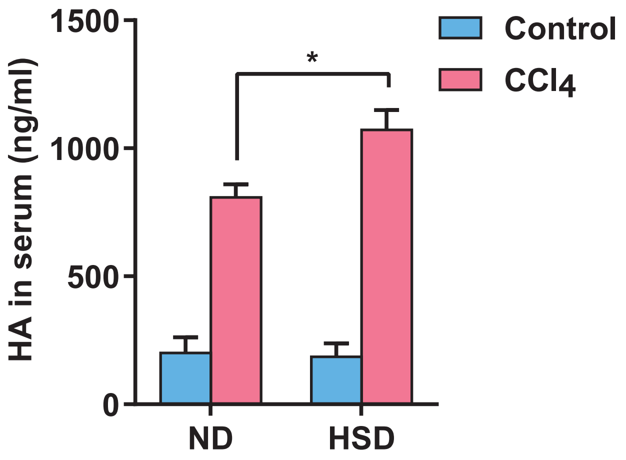

Supplementary figure 1 The detection of serum biochemical HA.

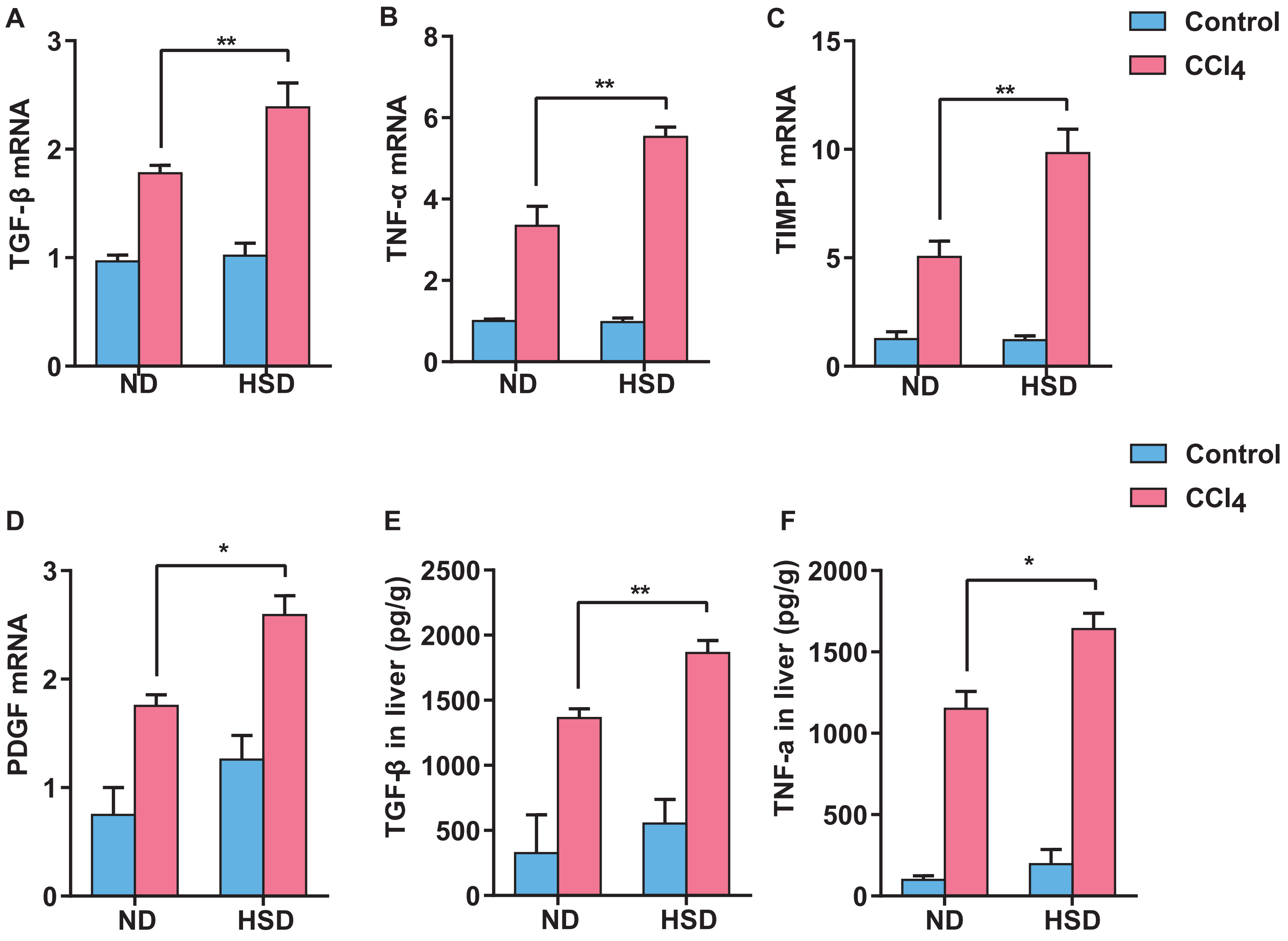

Supplementary figure 2 Relative expression of Inflammatory cytokines mRNA in liver.

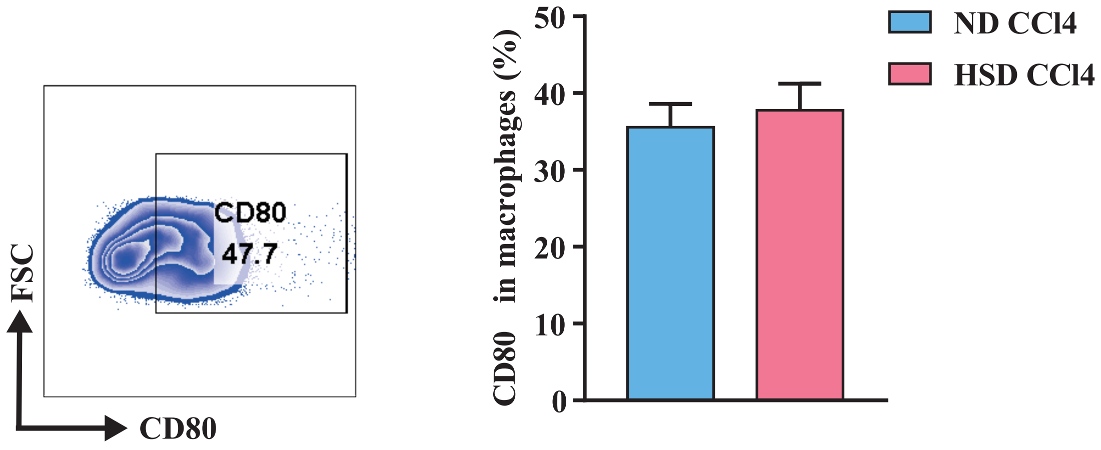

:

Supplementary figure 3

Left: Representative flow cytometry images of CD80 expression on macrophages. Right: Bar plots show the percentage of CD80 expression on macrophages in the liver.

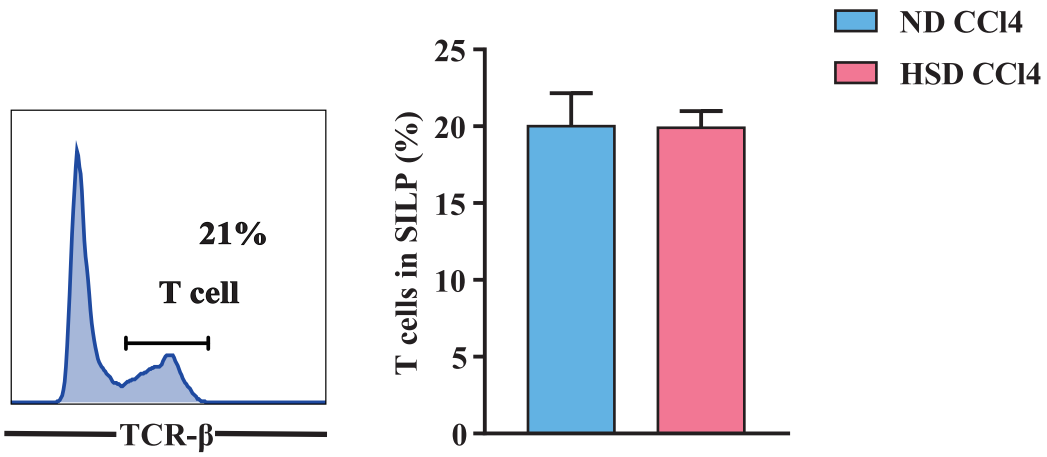

Supplementary figure 4

Left: Representative flow cytometry images of T cell in small intestine lamina propria. Right: Bar plots show the percentage of T cell in small intestine lamina propria.

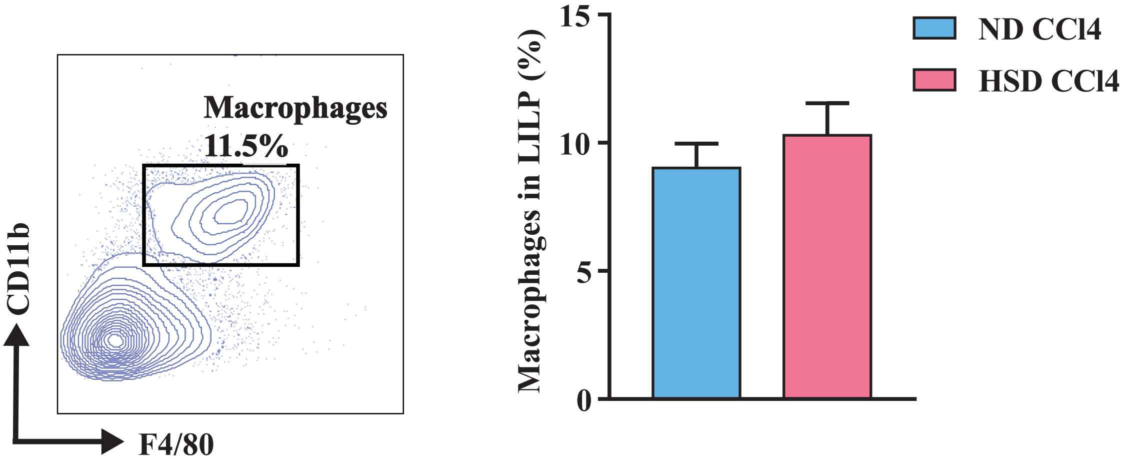

Supplementary figure 5

Left: Representative flow cytometry images of macrophages in large intestine lamina propria. Right: Bar plots show the percentage of macrophages in large intestine lamina propria.

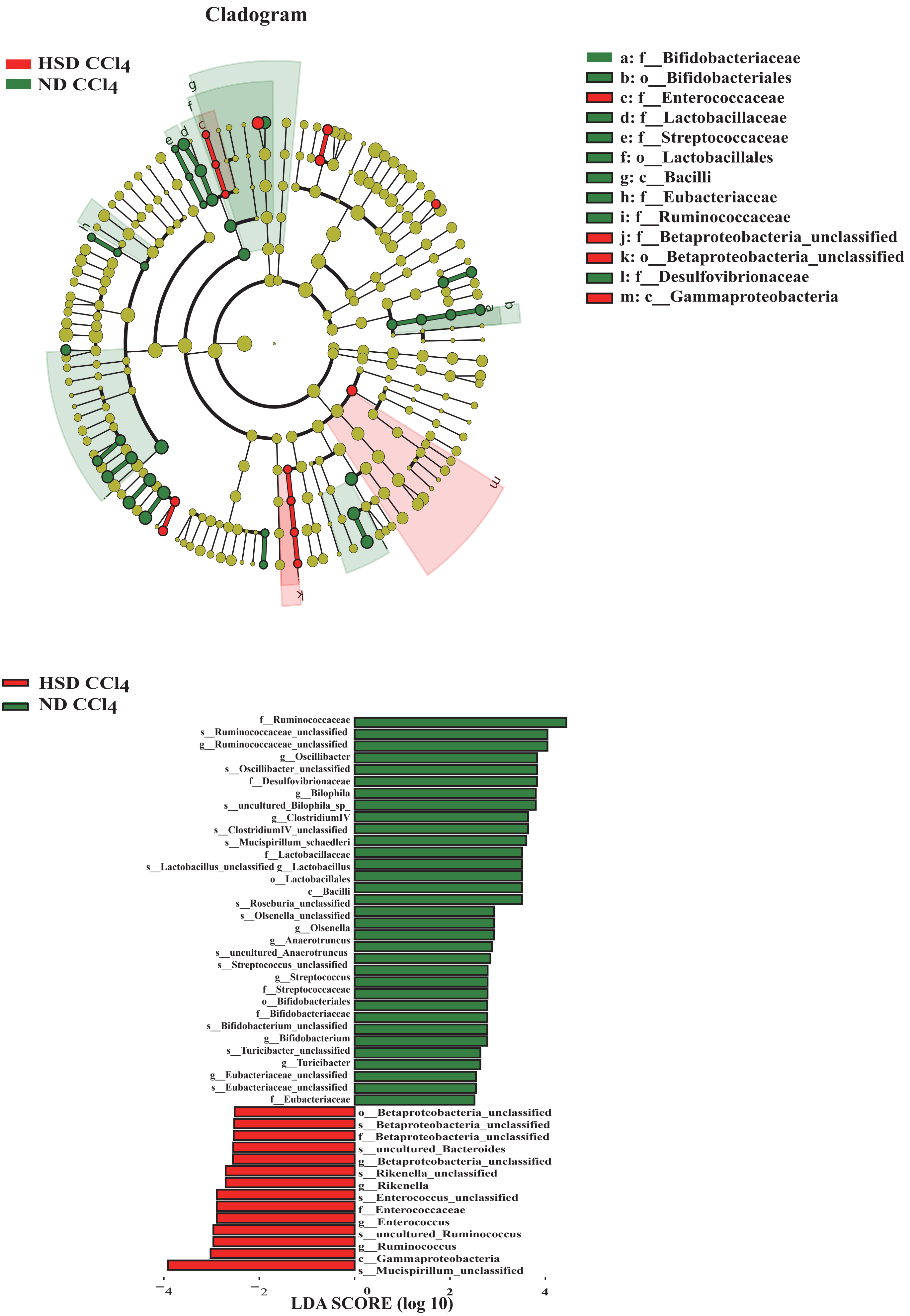

Supplementary figure 6 The characteristic biomarkers that differed between the ND CCL4 and HSD CCL4 groups.

A

B

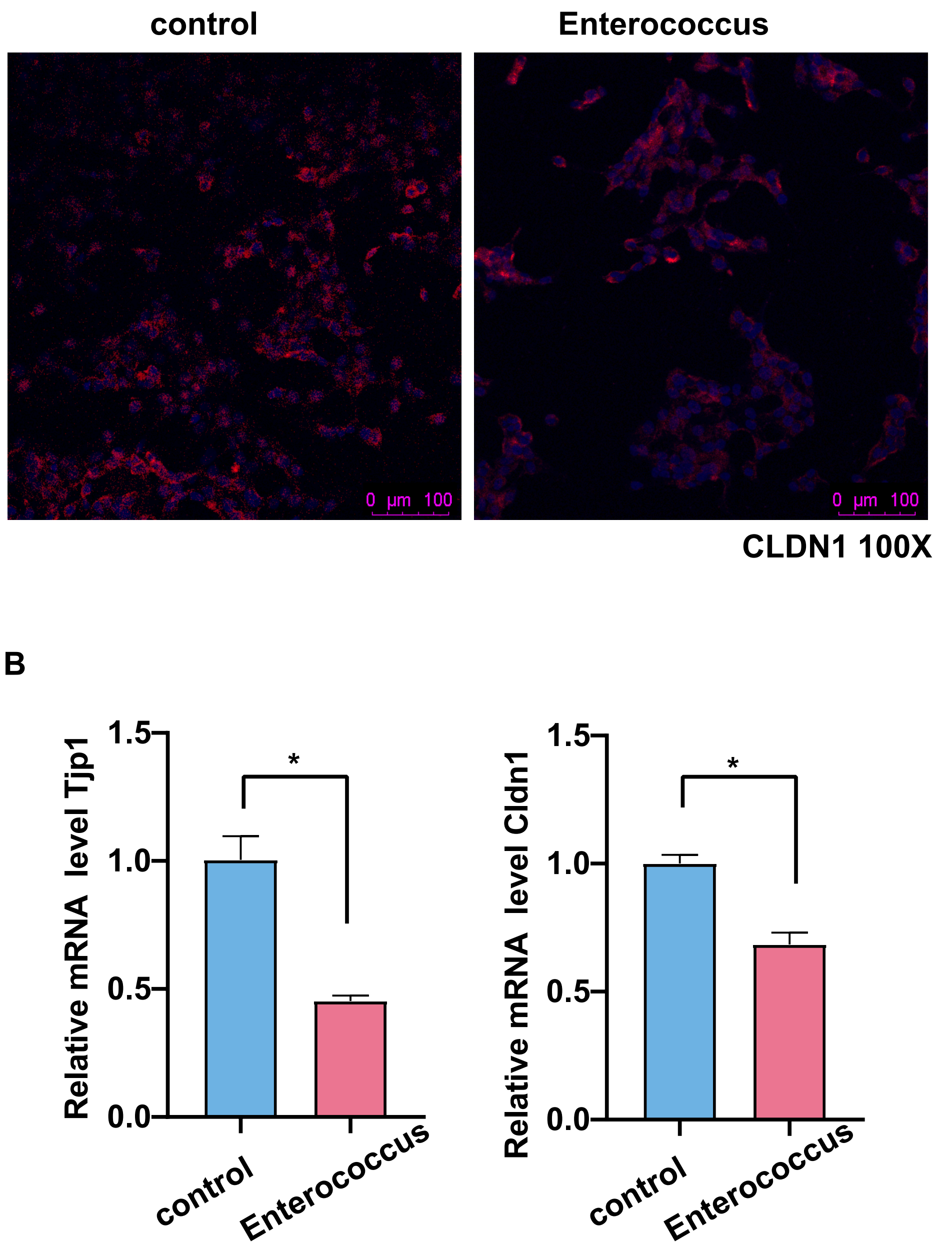

Supplementary figure 7 Immunofluorescence staining for CLDN1 (A) and relative expression mRNA of Tjp1 and Cldn1(B).

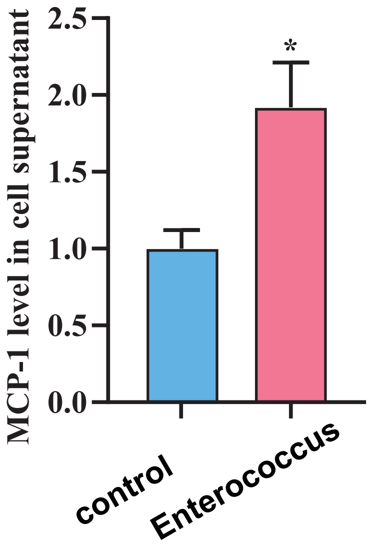

Supplementary figure 8 The releases of MCP-1 were detected in the culture supernatant.

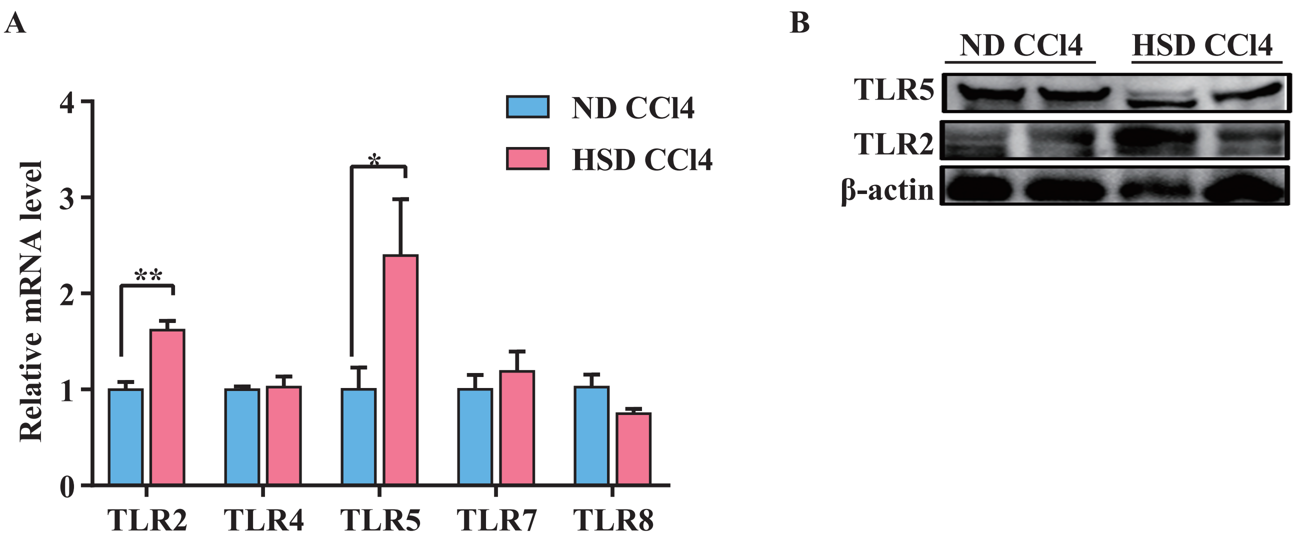

Supplementary figure 9 The TLRs expression in mice.

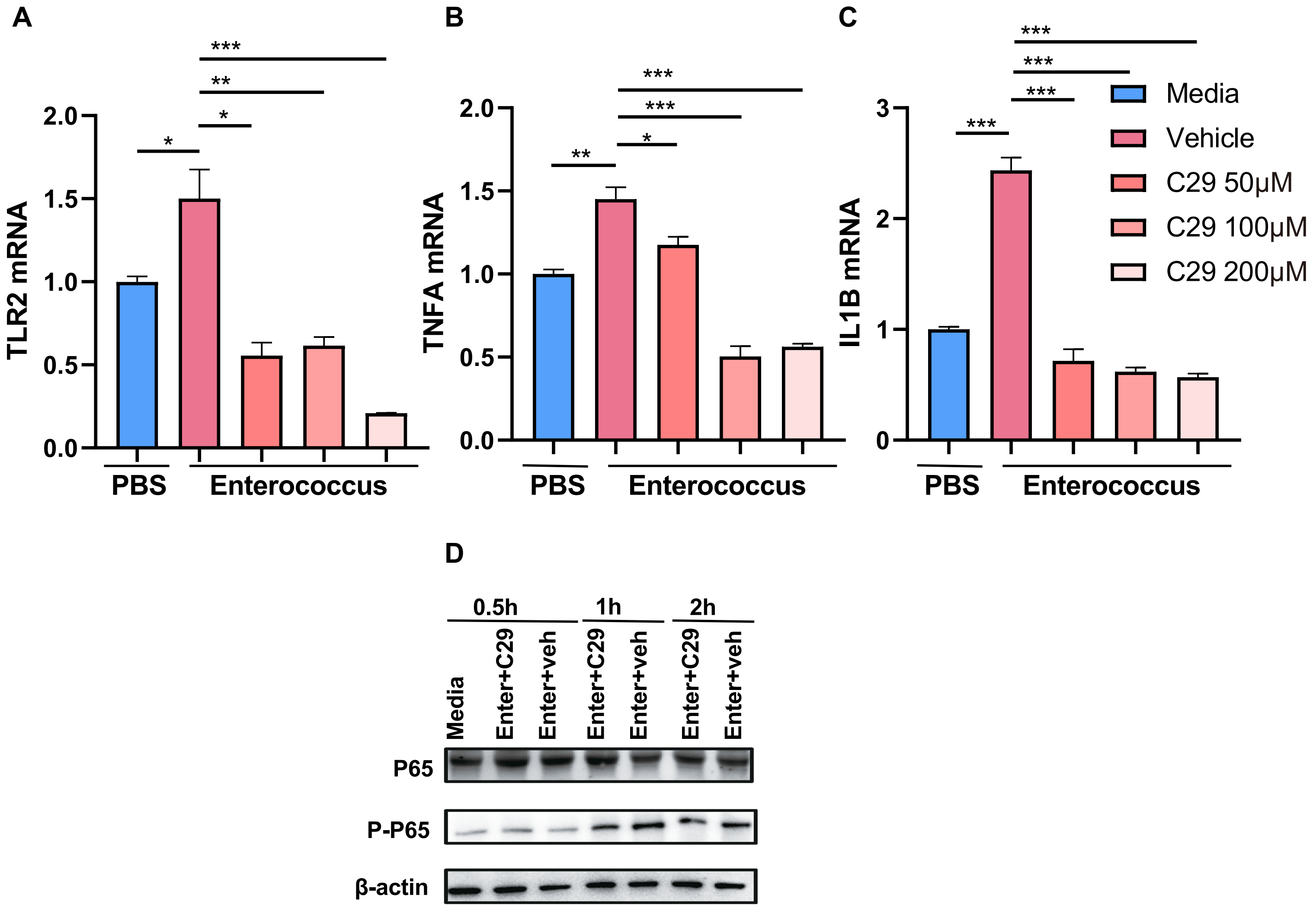

Supplementary figure 10 The model of TLR2 inhibition treated with C29.
TLR2,TNFA and IL1B production upon uptake in macrophages for 4h with media, vehicle (DMSO), or C29 (50 μM, 100 μM, or 200 μM) and then stimulated with Enterococcus stimulation(A-C).C29(50 μM) inhibited Enterococcus-induced p65 phosphorylation by Western blot(D).

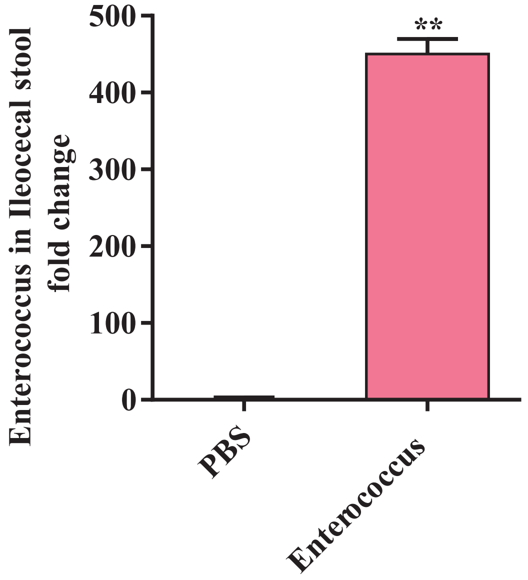

Supplementary figure 11 The Enterococcus content in feces.

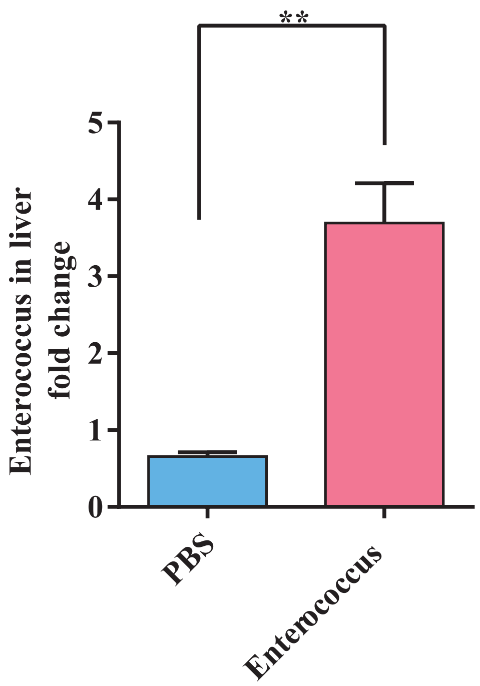

Supplementary figure 12 The Enterococcus content in liver.

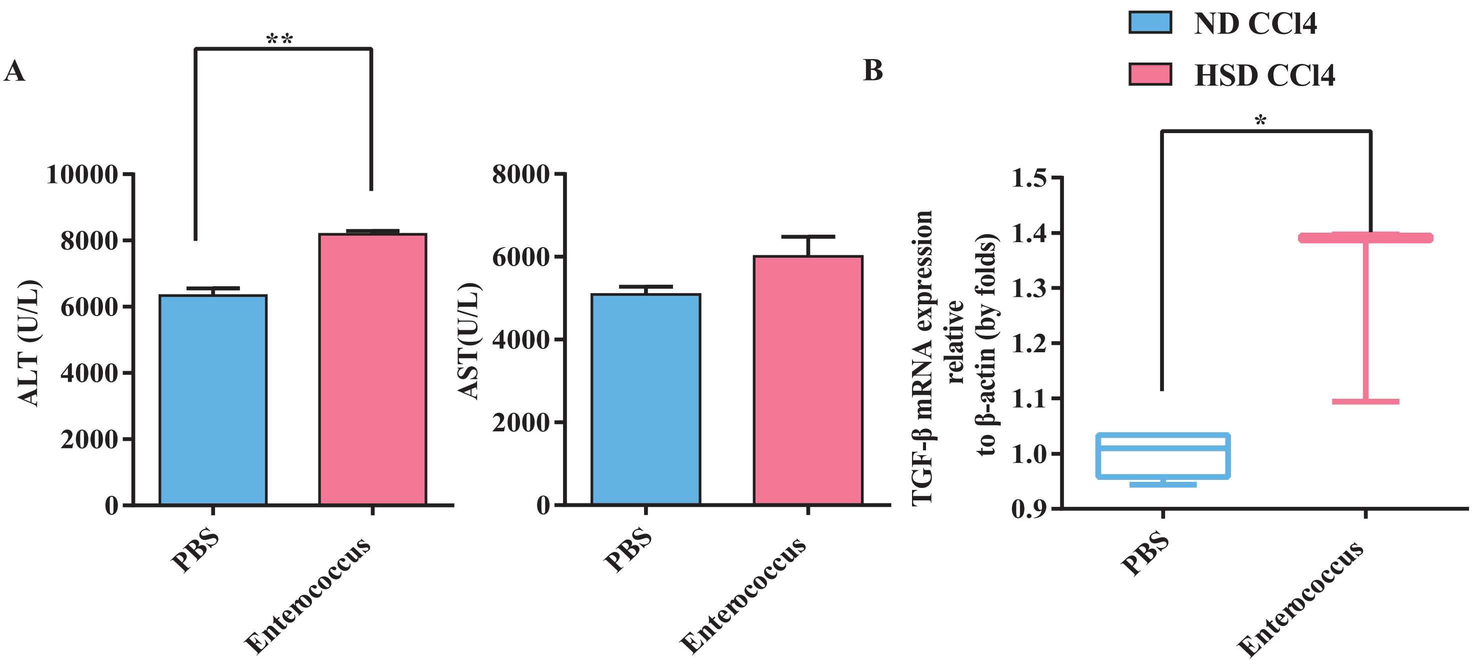

Supplementary figure 13 The release of ALT and AST in sreum(A). mRNA expression of TGF-β in liver(B).
